# FDG-PET/CT pitfalls in oncological head and neck imaging

**DOI:** 10.1007/s13244-014-0349-x

**Published:** 2014-08-26

**Authors:** Bela S. Purohit, Angeliki Ailianou, Nicolas Dulguerov, Christoph D. Becker, Osman Ratib, Minerva Becker

**Affiliations:** 1 Department of Imaging, Division of Radiology, Geneva University Hospital, Rue Gabrielle Perret Gentil 4, 1211 Geneva 14, Switzerland; 2 Department of Otorhinolaryngology, Head and Neck Surgery, Geneva University Hospital, Rue Gabrielle Perret Gentil 4, 1211 Geneva 14, Switzerland; 3 Department of Imaging, Division of Nuclear Medicine, Geneva University Hospital, Rue Gabrielle Perret Gentil 4, 1211 Geneva 14, Switzerland

**Keywords:** Positron emission tomography-computed tomography (PET/CT), Pitfalls, Head and neck tumours

## Abstract

**Objectives:**

Positron emission tomography-computed tomography (PET/CT) with fluorine-18-fluorodeoxy-D-glucose (FDG) has evolved from a research modality to an invaluable tool in head and neck cancer imaging. However, interpretation of FDG PET/CT studies may be difficult due to the inherently complex anatomical landmarks, certain physiological variants and unusual patterns of high FDG uptake in the head and neck. The purpose of this article is to provide a comprehensive approach to key imaging features and interpretation pitfalls of FDG-PET/CT of the head and neck and how to avoid them.

**Methods:**

We review the pathophysiological mechanisms leading to potentially false-positive and false-negative assessments, and we discuss the complementary use of high-resolution contrast-enhanced head and neck PET/CT (HR HN PET/CT) and additional cross-sectional imaging techniques, including ultrasound (US) and magnetic resonance imaging (MRI).

**Results:**

The commonly encountered false-positive PET/CT interpretation pitfalls are due to high FDG uptake by physiological causes, benign thyroid nodules, unilateral cranial nerve palsy and increased FDG uptake due to inflammation, recent chemoradiotherapy and surgery. False-negative findings are caused by lesion vicinity to structures with high glucose metabolism, obscuration of FDG uptake by dental hardware, inadequate PET scanner resolution and inherent low FDG-avidity of some tumours.

**Conclusions:**

The interpreting physician must be aware of these unusual patterns of FDG uptake, as well as limitations of PET/CT as a modality, in order to avoid overdiagnosis of benign conditions as malignancy, as well as missing out on actual pathology.

***Teaching points*:**

• *Knowledge of key imaging features of physiological and non-physiological FDG uptake is essential for the interpretation of head and neck PET/CT studies.*

• *Precise anatomical evaluation and correlation with contrast-enhanced CT, US or MRI avoid PET/CT misinterpretation.*

• *Awareness of unusual FDG uptake patterns avoids overdiagnosis of benign conditions as malignancy.*

## Introduction

Positron emission tomography-computed tomography (PET/CT) with fluorine-18-fluorodeoxy-D-glucose (FDG) plays a major role today in the pre-therapeutic work-up and post-therapeutic monitoring of patients with head and neck tumours. FDG-PET/CT is now routinely used in the head and neck for the delineation of the primary tumour, detection of regional nodal metastases, distant metastases and second primary tumours. Further indications include assessment of post-treatment response, long-term surveillance to detect recurrence and, last but not least, detection of an unknown primary tumour [1–5]. Thus, FDG-PET/CT tremendously facilitates the management of head and neck cancer patients in whom treatment is often expensive and associated with a significant morbidity [1–4]. However, the interpretation of FDG-PET/CT studies in the head and neck may be quite challenging due to the inherently complex anatomy, physiological variants and unusual patterns of FDG uptake after radiation therapy and surgery [1–4, 6–9]. Because FDG is not a tumour-specific tracer, it can accumulate in a variety of benign processes including benign tumours, inflammatory, post-traumatic and iatrogenic conditions. Benign non-physiological FDG uptake may be seen in up to 25 % of whole-body PET/CT examinations, and FDG uptake may mimic malignant tumours in more than half of these lesions [10]. Lesion characterisation on the CT portion of the PET/CT study is therefore of utmost importance as it increases the specificity of PET/CT reporting [10]. Although FDG interpretation pitfalls are common in the head and neck, they have received only very limited attention in the literature. The purpose of this review is to discuss the most common interpretation pitfalls in the purview of head and neck PET/CT and how to avoid them. The added value of contrast-enhanced CT (CECT) and/or magnetic resonance imaging (MRI) with diffusion-weighted imaging (DWI) sequences or ultrasound (US) to solve diagnostic dilemmas is equally discussed.

## PET/CT imaging protocols

A detailed discussion of institutional PET/CT protocols used in head and neck cancer patients is beyond the scope of this article. Nevertheless, familiarity with the basic principles of PET/CT imaging in head and neck oncology is important as biological factors and the choice of imaging parameters, such as field of view (FOV), slice thickness or use of iodinated contrast material may influence the interpretation of PET/CT findings. In most institutions, head and neck tumour patients will often undergo a standard PET/CT examination without intravenous contrast material; the area investigated typically extends from the mid-forehead to the mid-thigh. Nevertheless, an increasing number of authors advocate the additional use of a dedicated high-resolution head and neck PET/CT (HR HN PET/CT) with small FOV, longer acquisition time per bed position and thinner slice thickness [11–13]. The additional use of intravenous contrast allows full diagnostic CT capability [11, 12] and improves diagnostic performance in the head and neck area [14]. HR HN PET/CT is of particular help in the identification of subtle morphological findings essential for the diagnosis of local tumour spread and for the detection of small lymph node metastases and recurrent disease, thus reducing false-negative PET/CT readings (see below). Routinely, PET/CT examinations are performed after a 6-h fasting period and when the measured intravenous serum glucose concentration prior to study begin is within normal limits (<200 mg/dl). In general, 150–555 MBq (about 5–6 MBq of F^18^-FDG per kilogram of body weight) [15] are administered intravenously and during the following 60-min uptake period patients are encouraged to rest and to refrain from talking or chewing PET/CT imaging of diabetic patients may be problematic because elevated glucose levels can cause competitive inhibition of FDG uptake in different tissues. Although intravenous insulin before FDG injection is effective in reducing glycaemia, it can cause increased FDG uptake in muscle and fat. Therefore, in patients with very high glucose levels (>200–250 mg/dl), scanning should be rescheduled if reasonably convenient [2]. A spiral CT scan for attenuation correction is obtained first, after which PET data are acquired. This unenhanced CT scan can be used not only for the attenuation correction of PET data but also for PET/CT image fusion thereby allowing precise lesion localisation. Typical whole-body CT parameters include: 120 kV, 240 mAs, 1.5 mm collimation, 40 cm FOV, soft tissue and bone window settings. PET data acquisition is usually started after CT with 3–6 min per bed position for a total of seven to nine beds covering the area from the mid-forehead to the proximal thigh. Typical whole-body PET parameters include: 168 PET matrix, 4 iterations, 8 subsets, 4-mm pixel size. After the total body PET acquisition, an HR CECT can be obtained for the head and neck with the following parameters: 120 kV, 210 mAs, 0.64–0.75 mm collimation, 20–25 cm FOV (small FOV compared with whole-body PET/CT) soft tissue and bone window settings, intravenous bolus of 100 ml iohexol (CT acquisition started 1 min after intravenous bolus). For the head and neck area, PET acquisition parameters can be changed as follows: 6–12 min bed time, 256 PET matrix, 6 iterations, 4 subsets, 1.82-mm pixel size [11]. As suggested by several authors [11–13] and based on the experience in our institution, it is of utmost importance to obtain HR PET/CT images through the head and neck region with a separate acquisition from the body part to allow for detection of small lesions.

## Interpretation pitfalls

A variety of potential FDG-PET/CT interpretation pitfalls and artefacts can be observed on routine head and neck studies. The most common interpretation pitfalls are caused by variable physiological FDG uptake within Waldeyer’s ring, salivary glands, muscles or brown adipose fat (BAT) and by increased FDG uptake due to inflammatory and infectious conditions, recent surgery, previous chemoradiation, contralateral cranial nerve palsy and thyroid nodules with high glucose metabolism. Less often, lesions may be missed on PET/CT due to low FDG avidity, small lesion size or due to low scanner resolution. Common artefacts seen in the head and neck area are related to metallic implants and dental hardware or may occur due to patient motion between the CT and the PET acquisition. These interpretation pitfalls and artefacts, their pathophysiological mechanisms and key imaging features are discussed below. Special emphasis is put on how to avoid misinterpretation of findings and when additional imaging methods are of complementary value.

### Physiological FDG uptake in the head and neck

**Fig. 1:**
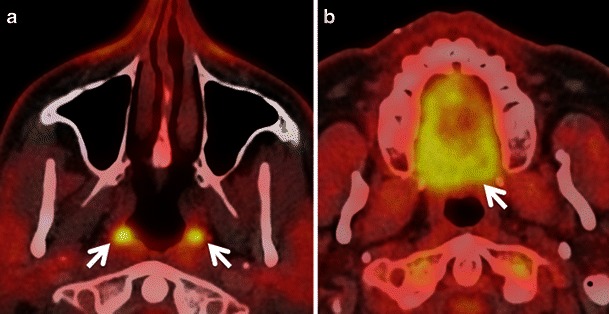
Axial PET CT images showing physiological symmetrical uptake in the nasopharyngeal tonsils (*arrows* in **a**) and in the mucosa of the hard palate and minor salivary glands beneath it (*arrow* in **b**)

Within the head and neck region, physiological FDG uptake (Fig. 1) is commonly seen in the mucosa of the soft and hard palate, Waldeyer’s ring, major salivary glands, minor salivary glands beneath the mucosa, extraocular muscles, neck, pharyngeal and laryngeal muscles, brown adipose tissue, thyroid gland and cerebral cortex [6–11, 15, 16].

#### Waldeyer’s ring

**Fig. 2:**
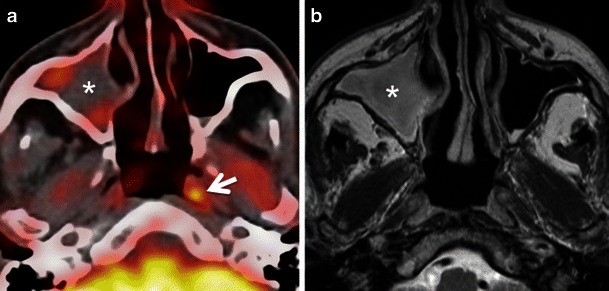
**a** Axial PET/CT image reveals asymmetrical FDG uptake in the nasopharynx (*arrow*) in a patient with SCC of the floor of the mouth. Opacification of the right maxillary sinus due to mucous retention (*asterisk*). **b** Axial T2-weighted MR image at the same level depicts symmetrical lymphoid tissue within the nasopharynx without underlying pathology. Endoscopy confirmed absence of tumour. Mucous retention in the right maxillary sinus (*asterisk*)

Low to moderate FDG uptake is normally seen in the lymphatic tissues of the Waldeyer’s ring (nasopharyngeal, palatine and lingual tonsils) due to FDG accumulation in macrophages and lymphocytes (Fig. 1). Physiological FDG uptake in lymphoid tissue can be symmetrical or asymmetrical. In patients with moderate and symmetrical FDG uptake, image interpretation is straightforward. Nevertheless, head and neck extranodal non-Hodgkin’s lymphoma (NHL) and squamous cell carcinoma (SCC) of the nasopharynx or base of the tongue may be occasionally bilateral making interpretation of images more difficult. In such cases it is imperative to look for associated anatomical abnormality on CT, such as presence of asymmetry, mass lesion, loss of fat planes and infiltration of surrounding facial deep spaces; all of which could be suspicious for tumour. Sometimes, asymmetrical focal uptake, which may occur as a physiological variant, can make interpretation of PET/CT images challenging (Fig. 2a). Since the Waldeyer’s ring is a common site for the origin of NHL and primary SCC of the head and neck, asymmetrical FDG uptake by normal lymphoid tissue may act as a confounding factor in the identification of these tumours. Detailed anatomical evaluation by CECT or MRI (Fig. 2b) and clinical evaluation including endoscopy with biopsy may be necessary to reach the correct diagnosis [1–4, 6–9]. In the absence of anatomic abnormality, some authors have suggested that ratios of standardised uptake values (SUVs) of the normal to abnormal side may help in reaching the diagnosis. For example, some authors describe a statistically significant (*P* < 0.001) SUV difference between benign uptake (3.0 ± 1.16) and malignant uptake (7.03 ± 3.83) in the nasopharynx [16]. Also, SUV ratios of lateral nasopharyngeal recess uptake to the palatine tonsil are significantly lower in benign compared with malignant lesions and may be used to differentiate nasopharyngeal cancer from physiological FDG uptake [16]. Similarly, the mean SUVmax ratio of the two tonsils may be used as an accurate biomarker to differentiate between tonsillar SCC and physiological FDG uptake [17]. Davidson et al. [17] found that in patients with tonsillar cancer, the mean difference in SUVmax between tonsils was 10.43 ± 7.07, which was significantly greater than that in control subjects (0.62 ± 0.54; *P* < 0.0001 and the mean SUVmax ratio between tonsils in patients with carcinoma was threefold higher than in control subjects (3.79 ± 1.69 vs 1.18 ± 0.13; *P* < 0.0001).

#### Salivary glands

**Fig. 3:**
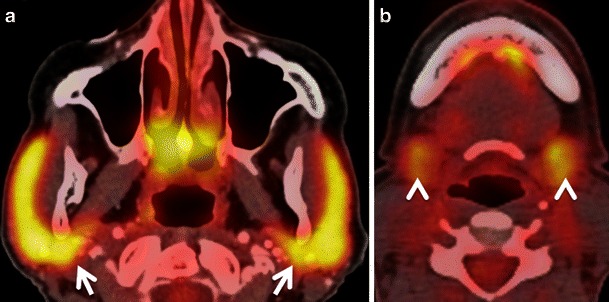
Axial PET/CT images obtained at the level of the parotid glands (**a**) and submandibular glands (**b**). Physiological symmetrical and high FDG uptake in both parotid glands (*arrows*) without underlying pathology. Physiological slightly asymmetrical and moderate FDG uptake in bilateral submandibular glands (*arrowheads*) and sublingual glands without underlying pathology

**Fig. 4:**
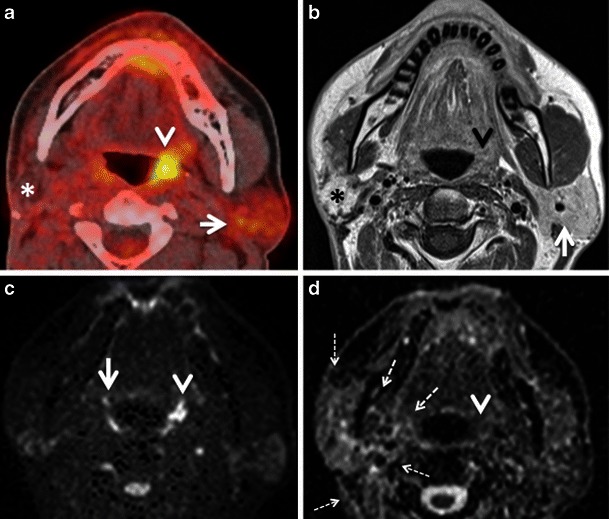
**a** Axial PET/CT image demonstrates asymmetrical FDG uptake of the tonsillar fossae (*arrowhead*) and parotid glands (increased uptake on the left, *arrow*) in a patient previously irradiated for SCC of the right base of the tongue with sparing of the left side. Note increased FDG uptake of the non-irradiated left parotid gland (*arrow*) and fatty infiltration of the irradiated right parotid gland (*asterisk*). **b** Corresponding axial contrast-enhanced T1-weighted image shows no pathological findings in the left tonsillar fossa (*arrowhead*) and left parotid gland (*arrow*). Note atrophy and fatty infiltration of the right parotid gland (*asterisk*). **c** Corresponding *b*1,000 DW MR image reveals physiological hyperintensity in the tonsillar fossae more pronounced on left (*arrowhead*) compared with the right (*arrow*) due to normal lymphoid tissue on the left and atrophy of lymphoid tissue on the right. **d** Corresponding ADC map showing physiologically low ADC values on the left (*arrowhead*) due to normal lymphoid tissue. *Dashed arrows* point at radiation port on the right

FDG is physiologically taken up by the salivary glands and excreted into saliva. Low to high FDG symmetrical uptake is noted in the parotid and submandibular glands (Fig. 3). Benign conditions like sarcoidosis, tuberculosis, viral infections, bacterial infections, obstructive lithiasis and radiation-induced sialadenitis can also cause increased FDG uptake in the salivary glands. Asymmetrical salivary gland uptake in the floor of the mouth can mimic focal areas of oral cavity malignancy. Asymmetrical submandibular gland uptake can be seen in patients who have undergone surgical removal of a gland with contralateral hypertrophy and in patients who have undergone unilateral radiation therapy [2–4, 6–9] (Fig. 4). This focal asymmetrical uptake may occasionally mimic metastatic adenopathy at level IB or II [4]. While symmetrical diffuse uptake in the salivary glands may be physiological (Fig. 3), focal, asymmetrical uptake may also be suggestive of FDG-avid benign or malignant salivary gland tumours like Warthin tumour, pleomorphic adenoma, primary parotid lymphoma or intraparotid lymph node metastasis from skin cancer. The SUVs of most malignant parotid tumours are significantly higher than those of benign lesions. However, some benign tumours may show very high FDG uptake (typically Warthin tumours), thereby mimicking malignant disease, while a few malignant parotid gland neoplasms (typically adenoid cystic carcinomas, low grade mucoepidermoid carcinoma or necrotic SCC) may have no significant FDG avidity [2, 4, 9, 18–20]. Also, some studies show that high-grade salivary gland malignancies are associated with higher SUVs compared with low grade malignancies [20]. Smaller salivary gland tumours may be missed on CECT. Hence, in the presence of a focal asymmetrical FDG uptake, further evaluation with ultrasound (US)/US-guided fine needle aspiration cytology (US FNAC) and/or MRI is often mandatory to reach the definitive diagnosis [7, 18–20]. Due to wide availability, low cost and high diagnostic accuracy, US is commonly used as a problem-solving modality for the evaluation of salivary gland conditions [21–23]. Many authors advocate the use of US FNAC for the pre-operative evaluation of parotid masses due to its good diagnostic accuracy. One study [24] shows that the positive predictive value of US FNAC for diagnosing parotid malignancy was 84.6 % and the negative predictive value was 96.4 %. Although MRI is mostly used for the precise assessment of tumour spread, it can also be used as an adjunct to differentiate between benign and malignant salivary gland tumours. Malignant salivary gland tumours are often associated with ill-defined margins, low T2 signal due to high cellularity, perineural spread most often along the facial nerve (when originating in the parotid gland), invasion of the mandible, skull base, subcutaneous fat or skin and lymph node metastases [25]. Nevertheless, these signs may be absent in small-sized or well-differentiated malignant tumours, making differentiation from benign lesions difficult. Quantitative analysis with DWI may be of help as the mean apparent diffusion coefficient (ADC) of benign tumours (1.72 × 10^−3^ mm^2^/s is often higher than that of malignant tumours (1.05 × 10^−3^ mm^2^/s (*P* < 0.001) [26], with the exception of Whartin tumours, which typically show very low ADC values.

#### Muscles

**Fig. 5:**
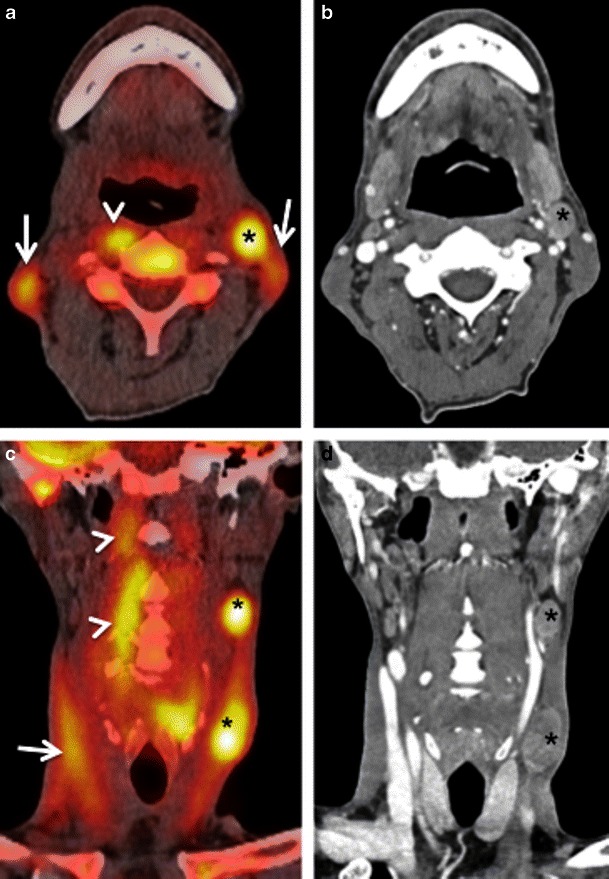
HIV positive patient with SCC of the left piriform sinus. **a** Axial PETCT illustrates a nodular area of high FDG uptake at left level II (*asterisk*) and bilateral nodular areas of symmetrical high FDG uptake laterally in the neck (*arrows*). There is an additional nodular area of high FDG uptake in the right retropharyngeal region (*arrowhead*). All nodular areas of high FDG uptake appear to represent metastatic adenopathy. **b** Corresponding axial CECT image shows an enlarged enhancing left level II node (*asterisk*). No suspicious adenopathy is seen at the other sites of increased FDG uptake including the lateral sides of the neck as well as the right retropharyngeal region. **c** Corresponding coronal PET/CT image confirms the linear nature of the foci of high FDG uptake in both sternocleidomastoid muscles (*arrow*) and in the right longus colli muscle (*arrowheads*). Areas of avid nodular FDG uptake are seen at left level II and III (*asterisks*). **d** Corresponding coronal CECT image confirms that the linear areas of FDG uptake correspond to neck muscles. Metastatic cervical adenopathy is confirmed at left level II and III (*asterisks*). The high uptake in the right longus colli and bilateral sternocleidomastoid muscles was related to involuntary contraction in this very anxious patient

Physiological FDG uptake is often seen in the muscles of the head and neck, which can constitute a diagnostic dilemma in the interpretation of PET scans [2–4, 6–9]. Prominent physiological uptake can be seen in the tongue and in the pterygoid muscles on vocalisation and chewing after FDG injection. Prominent FDG uptake is also often seen in the extraocular muscles due to eye motion. In the neck, physiological FDG uptake can be seen both in the visceral and non-visceral compartment musculature. In the visceral compartment, pronounced uptake in the cricopharyngeus and posterior cricoarytenoid muscles on phonation can interfere with the interpretation of PET scans in patients with hypopharyngeal, oesophageal and thyroid cancers, in whom this physiological uptake may mimic pathology [2, 4, 6, 27]. Uptake in the anterior portion of the genioglossus muscles can mimic or obscure small floor of the mouth cancers. Contraction-induced increased FDG uptake in the cervical muscles, strap muscles and paraspinal muscles in anxious patients (in particular sternocleidomastoid, scalenus anterior, longus colli, longus capitis and inferior obliquus capiti muscles) can mimic lymph node metastasis or, alternatively, may lead to false-negative findings obscuring disease truly present in underlying lymph nodes [2–4, 6–9, 28, 29]. Uptake in the anterior scalenus muscle mimicking supraclavicular lymph node metastasis in a case of lung cancer has been described [29]. Muscle uptake is generally linear and can be traced from the origin to insertion on fused PET/CT images (Fig. 5) Therefore, careful analysis of two-dimensional (2D) multiplanar reconstructions in the coronal and sagittal planes is mandatory whenever findings are unclear on axial PET/CT images [2, 4, 7–9, 28]. The administration of benzodiazepines before FDG injection helps to decrease muscle uptake; however, it is rarely done in clinical routine. Also, patients may be advised to stay relaxed and avoid talking, eating and chewing after the injection of FDG [7–9, 28]. A further factor influencing FDG uptake in muscle is insulin. Insulin administration prior to FDG PET/CT leads to increased accumulation of FDG in muscle, degrading image quality and hampering correct image interpretation [2].

#### Brown adipose tissue (BAT)

**Fig. 6:**
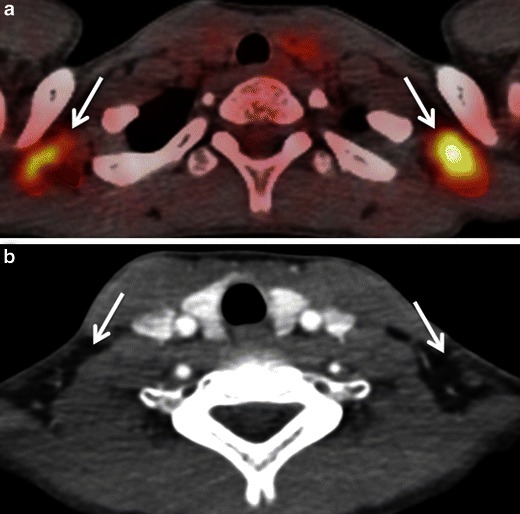
**a** Axial PET/CT image depicts bilateral symmetrical areas of avid FDG uptake in the supraclavicular regions (*arrows*). These may be confused for metastatic supraclavicular adenopathy. **b** Corresponding axial CECT image detects no focal abnormality in the supraclavicular regions (*arrows*). Note that the areas of high uptake seen in **a** correspond to low attenuation fatty tissue (*arrows*) in **b**

BAT can occur anywhere in the neck. It is, however, most often encountered in the lower neck and upper mediastinum. High physiological FDG uptake in BAT may sometimes mimic metastases (Fig. 6). Sympathetic stimulation stimulates brown adipocytes and increases their metabolic activity, leading to increased FDG uptake. A typical finding of BAT-related FDG uptake is symmetrical FDG uptake in the supraclavicular, mid-axillary, paraspinal and posterior mediastinal regions. These areas show fat attenuation (−50 to −150 Hounsfield units [HU]) on the corresponding CT images. Precise PET/CT image fusion, careful analysis of CT images and knowledge of human BAT distribution help to avoid misdiagnosis of brown fat deposits for pathology [2, 4, 6–9, 30]. Hypermetabolic BAT is more commonly seen in children than in adults and is more prevalent in females than in males. It occurs more frequently in patients with low body mass index and in cold weather [30]. Some authors describe variations in supraclavicular BAT FDG uptake in breast cancer patients after chemotherapy with drugs like docetaxel [31]. Hibernomas are benign fatty tumours arising from vestigeal fetal brown fat and are usually seen in the neck, back and mediastinum. These benign lesions often show intense FDG uptake, thereby mimicking soft tissue sarcomas [32]. FDG uptake in brown fat can be reduced pharmacologically with beta-blockers and benzodiazepines, if necessary [6–8].

### Increased FDG uptake potentially causing false-positive findings

#### Inflammation and infection

**Fig. 7:**
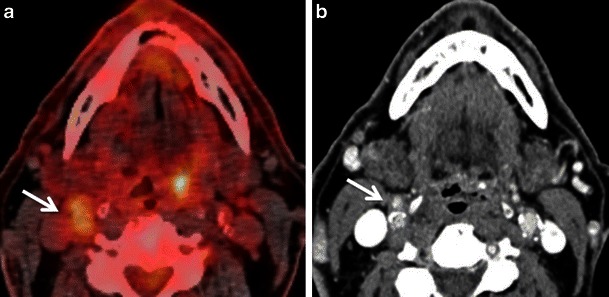
**a** Axial PET/CT image depicts a focal nodular area of high FDG uptake at right level II (*arrow*) and at the left base of tongue (*arrowhead*). This patient was a follow-up case of SCCof the right floor of mouth. The nodular FDG uptake at right level II was considered suspicious for nodal recurrence whilst the focal FDG uptake at the left base of tongue was ascribed to probable lymphoid tissue uptake. **b** Corresponding axial CECT image detects no evidence of metastatic cervical adenopathy at right level II. The focal FDG uptake at right level II corresponds to partly calcified plaques in the right internal and external carotid arteries (*arrow*). Follow-up confirmed no lesion in the left base of the tongue

As mentioned above, benign conditions like inflammation, infection and granulomatous diseases may show increased FDG uptake in head and neck cancer patients, thereby mimicking malignancy. The uptake in these cases is attributed to increased glycolysis in activated inflammatory cells (mainly macrophages). Increased FDG uptake is typically seen at sites of infection, indwelling ports and catheters, foreign body granulomas, reactive nodes, vasculitis and in active atherosclerotic plaques (Fig. 7), [1, 3, 4, 7–10, 33–37]. Recent studies have shown that FDG uptake not only allows reliable identification of vulnerable plaques in the carotid arteries but also reflects the severity of atherosclerotic vessel wall inflammation [36]. Therefore, precise image fusion between PET and CT data sets, as well as correlation with CECT, is necessary to avoid misdiagnosis of carotid plaque uptake as metastatic lymph nodes (Fig. 7). This pitfall is typically encountered when only unenhanced CT scans are available for image interpretation and whenever patient motion between the CT and the PET acquisition leads to mis-coregistration of images (see below).

Often the clinical history points to the cause of FDG uptake; however, inflammatory conditions (such as periodontal disease, dental infection, active atherosclerotic plaques or tuberculosis) and neoplastic disease can coexist. Reactive nodes are commonly encountered in the head and neck, and may result in a decreased specificity of FDG-PET/CT for the nodal staging of head and neck cancers. Precise image fusion, correlation of PET with morphological CT findings and 2D multiplanar reconstructions are essential in order to avoid overdiagnosis of benign conditions as malignancy, as well as missing out on actual pathology. Evaluation of SUVs may help to differentiate between benign and malignant disease, whereby SUVs greater than 3 are considered as a general guide to indicate neoplasm. Nevertheless, SUVs greater than 3 can also occur in acute infection, such as sinusitis, osteomyelitis, suppurative lymphadenopathy and abscess. If diagnostic dilemma still exists, sequential follow-up imaging, US examination of the neck and, at times, US FNAC may be needed [1, 3, 4, 7–9, 33, 34, 38, 39].

**Fig. 8:**
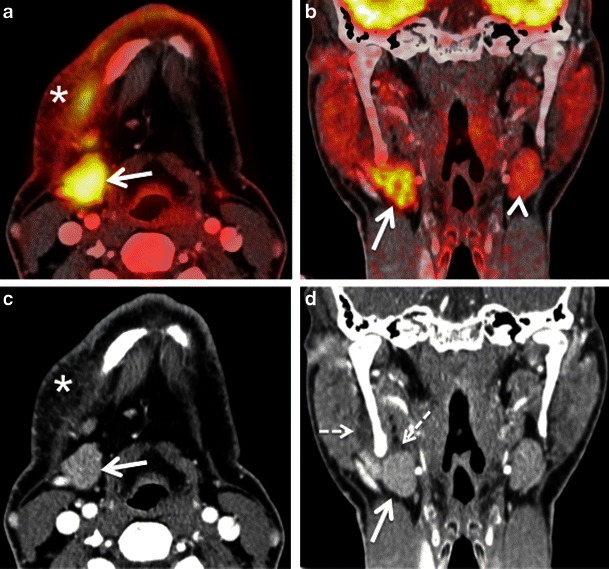
Axial (**a**) and coronal (**b**) PET/CT images illustrate high FDG uptake of the right submandibular gland (*arrows*) and soft tissues of the neck (*asterisk*) in a patient investigated for histiocytosis. The left submandibular gland (*arrowhead*) shows moderate FDG uptake. Note that the hypermetabolic submandibular gland can be easily mistaken for lymphadenopathy unless coronal images are carefully analysed. Corresponding axial (**c**) and coronal (**d**) CECT images reveal slightly increased enhancement of the right submandibular gland (*arrows*), reticulated aspect of subcutaneous fatty tissue (*asterisk*) and thickening of the right platysma muscle due to sialadenitis with phlegmon. Dashed arrows in d point at the phlegmon extending cranially in the masticator space. US revealed lithiasis as the cause of sialadenitis

Other than osteomyelitis and reactive nodes, inflammation of salivary gland parenchyma and of salivary ducts can constitute a diagnostic pitfall (Fig. 8). Increased salivary gland uptake can be seen in obstructive as well as non-obstructive sialadenitis. In obstructive sialadenitis, the increased FDG uptake can be explained by the accumulation of excreted FDG in the dilated ductal system. The FDG uptake seen in non-obstructive sialadenitis is caused by activated white blood cells, increased levels of glucose transporters (mainly GLUT 1 and GLUT 3), cytokines and growth factors [10]. Careful correlation of FDG uptake with morphology, as depicted on the CT or CECT part of the PET CT is essential for diagnosis (Fig. 8). CECT findings in sialadenitis include major enhancement of gland parenchyma, reticulated aspect of peri-glandular fatty tissue due to phlegmon and, occasionally, the presence of a small abscess. CECT is less sensitive than US for the detection of early sialadenitis and sialolithiasis. Hypoechoic gland parenchyma, increased vascularisation on Doppler images and blurred gland margins are characteristic findings on US.

#### Inflammation caused by radiotherapy and chemotherapy

**Fig. 9:**
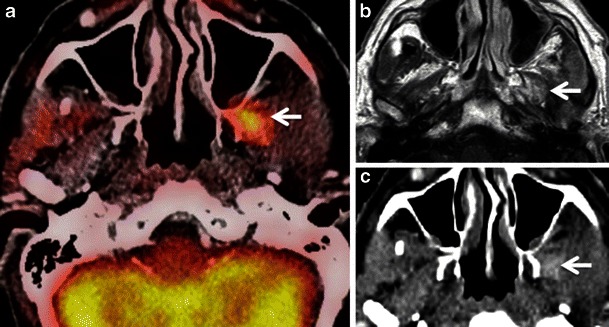
**a** Axial PET/CT image shows a hypermetabolic focus in the left infratemporal fossa (*white arrow*). This patient was a follow-up case of SCC of the left retromolar trigone, 6 months post radiotherapy, presenting with left V3 distribution pain and dysesthesia. **b** Corresponding axial contrast-enhanced T1W MR image demonstrates nodular architectural distortion with some enhancement (white arrow) in the left infratemporal fossa. **c** Corresponding axial CECT image reveals similar morphologic findings as the MR image: an ill-defined area of nodular enhancement (*white arrow*) in the left infratemporal fossa. The imaging differentials were tumour recurrence along left V3 nerve or post-radiotherapy immature (early) scarring. Surgery was negative for tumour recurrence and careful histologic analysis of the resected specimen yielded fibrotic scar tissue. Follow-up over a period of three years confirmed absence of recurrent disease

Chemoradiation is one of the mainstays in the treatment of head and neck cancers. FDG-PET is now routinely used for the differentiation of residual and recurrent tumours from post-radiotherapy changes, where anatomic imaging like CT or MRI can be difficult. FDG-PET/CT has a very high sensitivity (between 90 and 100 %) and very high negative predictive value (about 97 %) for the detection of tumour recurrence [1, 40–42]. However chemoradiation causes inflammation, oedema, hyperaemia, fibrosis and loss of tissue planes. The presence of post-treatment inflammatory tissue can cause increased FDG uptake, which may be confused for residual tumour (Fig. 9). There may also be considerable overlap in the SUVs of post-radiotherapy inflammatory tissue and tumour recurrence; in particular, regarding lymph node imaging. It is, therefore, recommended that follow-up PET/CT should be deferred for at least 2–3 months after chemo-radiotherapy to avoid false-positive results, with many authors advocating 12 weeks after treatment as the optimal timing [1, 3, 4, 9, 33, 34, 40–42].

Even if PET/CT is obtained after 12 weeks following completion of chemoradiation, false-positive findings may still occur. Post chemo-radiotherapy false-positive findings on PET/CT are caused by radiation-induced mucositis, reactive nodes, soft tissue necrosis and radionecrosis of bone [4, 8, 9, 33, 41]. CECT and MRI may also be equivocal in these situations. Radiation-induced mucositis is a common sequel of chemo-radiotherapy for oropharyngeal and laryngeal SCC and can persist for prolonged periods after treatment. Diffuse mild FDG uptake along the oropharyngeal and laryngeal walls are in keeping with inflammation; however, a more focal uptake should raise suspicion for ulceration or persistent disease. Osteoradionecrosis is a late complication of high-dose irradiation for squamous cell carcinoma of the oral cavity, pharynx and larynx, and constitutes a lifelong problem for cancer survivors. It can affect the jaws, larynx and hyoid bone, cervical spine, central skull base or temporal bone [43–45]. Although the risk of developing osteoradionecrosis has significantly diminished in recent years [44], osteoradionecrosis of the jaws and of the skull base still constitutes a common pitfall of FDG-PET/CT imaging [45, 46]. Often, osteoradionecrosis lesions show very high SUVs, equalling that of residual/recurrent tumour, and there is a significant overlap of SUVs in patients with osteoradionecrosis and tumour recurrence [46]. Correlation with CT images may help in some instances, as the prevalence of bony sclerosis is significantly more common in osteoradionecrosis, whereas recurrent tumours rather display solid or cystic soft tissue masses [46]. Nevertheless, in equivocal cases, a short-term follow-up PET/CT or MRI with diffusion-weighted sequences (DWI) may be additionally used for the differentiation between radiation-induced changes and residual/recurrent disease [4, 9, 33, 47]. Analysis of ADC values may help distinguish between residual cancer and benign post-treatment changes. Residual or recurrent SCCs show significantly lower ADC values than that of a benign post-treatment mass, with an ADC threshold value of around 1.3 × 10^−3^ mm^2^/s [47]. Due to their capability to obtain anatomical, functional and metabolic information in a single examination, hybrid PET/MRI systems hold promise to facilitate differentiation between radiation-induced changes and recurrent disease [5, 48–51]. Nevertheless, very little data are currently available regarding the clinical implementation of hybrid PET/MRI scanners in the head and neck [5, 48–51], and future studies will show whether PET/MRI outperforms PET/CT, DWI MRI or the combination of these techniques.

#### Inflammation caused by recent surgery

**Fig. 10:**
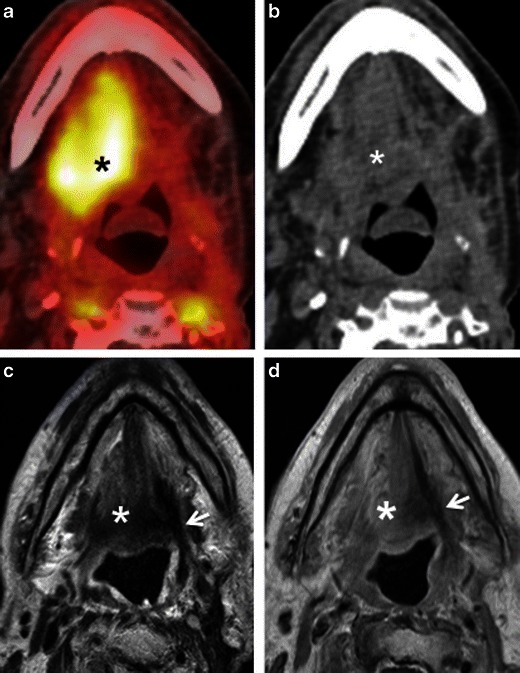
**a** Axial PET/CT image shows intense FDG uptake in the right root of the tongue and floor of the mouth (*asterisk*) in a patient with recent surgical resection of recurrent SCC of the left hemitongue. **b** Corresponding axial non-contrast CT image confirms post-surgical architectural distortion without an obvious underlying mass lesion (*asterisk*) on the right. Corresponding axial T2W (**c**) and contrast-enhanced T1W (**d**) images reveal a linear band of low signal intensity scar tissue on the left (*arrows*). No tumour is seen on the right (*asterisks*). Muscle architecture in the right floor of the mouth appears normal on T2 but there is some diffuse muscular enhancement on the corresponding T1W image. Compensatory hyperactivity and post-surgical inflammatory findings due to contralateral scarring were considered as the cause of this unusually high FDG uptake. Follow-up of 2 years confirmed absence of recurrence

Depending on institutional choices, surgery may be preferentially performed in patients with advanced nodal disease, in patients with oral cavity cancers or in patients with recurrent disease after chemoradiation. Although the morphological changes are straightforward after radical or functional neck dissection, partial glossectomy and total or partial laryngectomy, interpretation of the post-surgical neck may be more complicated in cases with reconstructive procedures using grafts or flaps (free or pedicle) and in cases with combined surgery and chemoradiation. Due to the confusing surgical anatomy, recurrent tumours are more easily identified with PET/CT than with CT alone due to the increased focal uptake of recurrent disease. Nevertheless, after surgical removal of a gland or muscle the contralateral gland or muscle may show increased FDG uptake (Fig. 10) mimicking tumour recurrence and careful analysis of postoperative neck changes can help to solve this diagnostic dilemma. Post-surgical inflammatory oedema, scarring and granulation tissue can also cause increased FDG uptake making the interpretation of PET/CT studies very difficult in particular if the CT part of the PET/CT study comprises only a low dose CT for attenuation correction or if no CECT images are available (Fig. 10). Granulation tissue is the first step in wound healing and develops from connective tissue around the damaged area [52, 53]. It mainly contains inflammatory cells, fibroblasts, myofibroblasts and small vessels [52]. Over time, myofibroblasts and small vessels gradually disappear and granulation tissue evolves into immature and then mature scar. The mechanism responsible for this process is granulation tissue apoptosis, which mainly occurs between 20 and 25 days after injury, the fibroblastic apoptotic cells being continuously removed by macrophages [52]. Histological and morphometric studies have shown that the transformation of granulation tissue into scar tissue usually takes place within the first 2 months of injury [52], However, the duration of this process may vary depending on the amount of damaged tissue. As this transformation is a continuous process, there is a smooth transition between granulation tissue, early (immature) scar with limited amount of collagen and late (mature) scar with significant collagen deposition. Major FDG uptake, as seen on PET/CT images, is typically seen during the first weeks to months after surgery; it tends to decrease gradually over time. Post-surgical anatomical distortion can further increase the diagnostic dilemma in such cases. Therefore, it is generally recommended that the follow up PET/CT be performed at least 4–6 weeks after surgery after acute inflammation has subsided. Detailed knowledge of the previous surgical procedure and surgical complications (abscess, phlegmon, flap necrosis) helps to avoid diagnostic errors. Occasionally, to make the PET/CT image fusion precise, an additional MRI may be necessary to avoid misdiagnosis [1–4, 8, 42] (Fig. 10).

#### Contralateral cranial nerve palsy

**Fig. 11:**
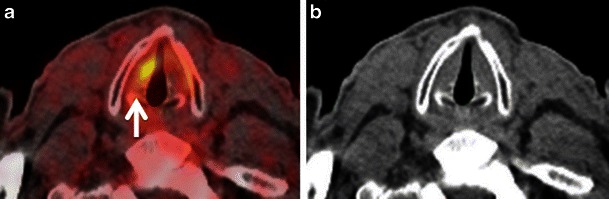
**a** Axial PET/CT image demonstrates a focal area of avid FDG uptake in the right true vocal cord (*arrow*) suspicious for a tumour lesion. **b** Corresponding non-contrast axial CT image shows no definite focal lesion in the right vocal cord. CT images at other levels of the larynx were normal. Clinical history mentioned recently diagnosed left vocal cord paralysis confirmed by fibre optic endoscopy. This compensatory increased uptake in the right vocal cord was due to recent left vocal cord paralysis. This case illustrates the need for rigorous correlation with clinical findings whenever the only abnormality observed is unilateral vocal cord FDG uptake

Asymmetrical FDG uptake in the vocal cords can be seen in patients with recurrent laryngeal nerve palsy from prior surgical intervention or tumour involvement or due to trauma. The compensatory activation of the non-paralysed vocal cord leads to increased metabolism and increased glucose consumption, especially in the thyroarytenoid muscle and in the posterior cricoarytenoid muscle; this increased metabolism is seen as a focal hypermetabolic spot on FDG PET images (Fig. 11). This imaging mimic may be confused with a tumour in the non-paralysed vocal cord unless CT images are carefully analysed [2, 4, 7, 8, 54–57]. On the contrary, if no FDG uptake is seen in both vocal cords and only in the posterior cricoarytenoid muscle, the interpreter may overlook vocal cord paralysis on FDG-PET/CT. Characteristic CT imaging findings of recurrent laryngeal nerve paralysis include paramedian position of the paralysed vocal cord, displacement of the ipsilateral arytenoid cartilage, compensatory medial rotation of ipsilateral aryepiglottic fold, dilated ipsilateral pyriform sinus and atrophy of posterior cricoarytenoid and thyroarytenoid muscles [4, 55, 57, 58]. PET/CT image fusion and clinical correlation can help to overcome this potential pitfall. History of hoarseness, prior surgery or radiation in the neck, larynx, thyroid or mediastinum may indicate injury to one of the recurrent laryngeal nerves [4, 55–57]. Laryngoscopic examination will help to confirm impaired movement of the contralateral vocal cord and also will rule out a primary pathology in the ipsilateral cord. However, any nodularity or apparent lesion in the vocal cord with focal FDG uptake is suspicious and warrants further evaluation [55–58].

Palsy of the hypoglossal nerve (XII), spinal accessory nerve (XI) or the mandibular division of the trigeminal nerve (V3) may also result in contralateral intense FDG muscle uptake. CT findings in long standing V3, XI and XII palsy are pathognomonic and usually do not require further imaging as long as the cause of nerve injury is known [59, 60]. In long standing XII palsy, CT reveals atrophy of the affected hemitongue with fatty infiltration, a clear-cut linear demarcation between the affected and non-affected muscles as well as tongue deviation. Prolapse of the paralysed hemitongue into the oropharynx when the patient is in the supine position is a further characteristic finding.

**Fig. 12:**
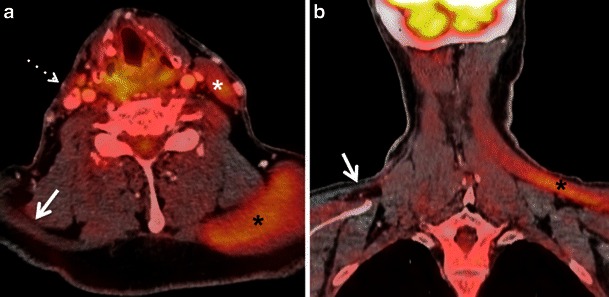
**a** Axial PET/CT image reveals compensatory FDG uptake of the left sternocleidomastoid muscle (*white asterisk*) and of the ipsilateral trapezius muscle (*black asterisk*) due to right spinal accessory nerve (XI) paralysis caused by a base skull adenocarcinoma. Also note atrophy of the right sternocleidomastoid muscle (*dotted arrow*) and of the right trapezius muscle (*arrow*). **b** Corresponding coronal PET/CT image depicts atrophy of the right trapezius muscle (*arrow*) and compensatory increased uptake of the left trapezius muscle (*black asterisk*)

In long-standing XI palsy, CT shows atrophy of the ipsilateral sternocleidomastoid and trapezius muscles (Fig. 12) and compensatory hypertrophy with focal or diffuse hypermetabolic FDG activity. In V3 palsy, fatty infiltration of the muscles of mastication (medial and lateral pterygoid, masseter and temporalis muscles), as well as of the tensor tympani, the anterior belly of the digastric and mylohyoid muscles are seen; contralateral muscle hypertrophy and increased FDG uptake are less often present at imaging.

In early muscle denervation due to recent palsy, muscle atrophy is not obvious on CT and contralateral compensatory FDG uptake may be mistaken for non-specific tracer accumulation or contralateral tumour in particular in the tongue [61]. Partial glossectomy can cause tongue fasciculations, which can lead to false-positive FDG uptake, thereby mimicking recurrent tumour [4, 33]. As MRI is more sensitive than CT for the detection of early signs of muscle denervation, correlation with MRI findings and with clinical data including electromyography helps to overcome this pitfall [4, 33].

#### Increased FDG thyroid gland uptake

**Fig. 13:**
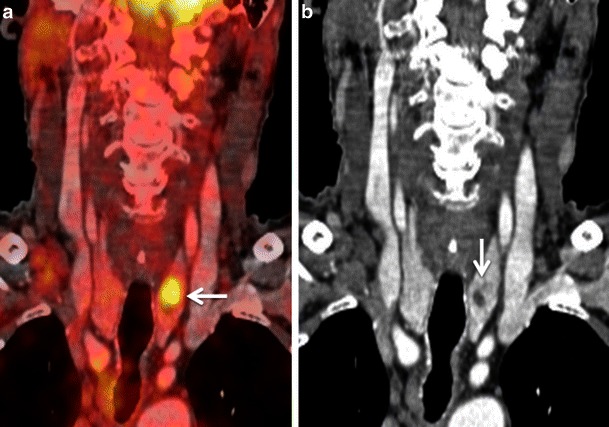
**a** Coronal PET/CT image illustrates a focal nodular area of high FDG uptake within the left thyroid lobe (*white arrow*), which can reveal an underlying malignancy in 25–50 % of cases. Therefore, US FNAC is recommended. **b** Corresponding coronal CECT image shows a small hypodense nodule within the left thyroid lobe (*white arrow*), corresponding to the focus of high FDG uptake on PET/CT. Subsequent US FNAC of the left thyroid nodule and follow-up over a period of 2 years yielded benign thyroid nodule, thereby indicating that the PET/CT result was false-positive for malignancy

The thyroid gland may show various appearances on PET studies with diffuse symmetrical FDG uptake, focal asymmetrical uptake or at times, no uptake [2, 6–8, 62, 63]. Various physiological, benign and pathological processes may be causative for these different patterns of FDG uptake. Mild diffuse symmetrical uptake may be seen in normal thyroid glands, in diffuse goitres or in autoimmune thyroiditis, whereas focal uptake may be seen in adenomatous nodules and thyroid malignancy [2, 6–8, 62, 63]. Although some authors state that the maximum SUV of malignant thyroid lesions is significantly higher than that of benign lesions (6.7 ± 5.5 vs 10.7 ± 7.8; *P* < 0.05) [63], the reported risk of malignancy in an area of focal FDG uptake in the thyroid ranges from 36–63 % [62, 63]. Therefore, all focal areas of moderate to intense FDG uptake in the thyroid gland should be further assessed with an US examination and US FNAC to exclude thyroid cancer (Fig. 13). Another potential imaging pitfall is the likely misdiagnosis of focal thyroid uptake as metastatic disease in the lower cervical nodal stations [64]. Precise image fusion helps to resolve this diagnostic dilemma [64]. However, in patients who have moved between the CT scan and the PET acquisition, it may be difficult to differentiate between metastatic level IV lymph nodes and small thyroid nodules, in particular in small-sized lesions. In our institution, we routinely perform an US examination of the neck in these situations and, if necessary, US FNAC.

### Factors potentially causing false negative PET/CT readings

#### Low FDG uptake, low image resolution and small lesion size

**Fig. 14:**
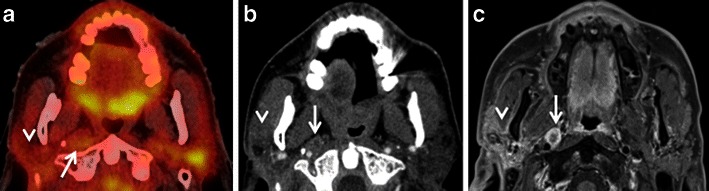
**a** Axial PET/CT image demonstrates slight asymmetrical uptake in the anatomic location of the longus colli muscles (*arrow*) in a patient previously treated by right partial parotidectomy and radiation therapy for a lymphoepithelial SCC of the right parotid gland. Note hypometabolism of the residual right parotid gland (*arrowhead*) and focal uptake in the left parotid gland. Corresponding axial CECT (**b**) and contrast-enhanced fat suppressed T1-weighted MR (**c**) images illustrate a necrotic metastatic retropharyngeal lymph node on the right side (*arrows*),mistaken on PET/ CT for minor prevertebral muscle activity and therefore yielding a false negative evaluation. Note the diffuse and heterogeneous enhancement of the remaining right parotid gland following surgery and radiotherapy (*arrowheads*) better depicted on MRI as on CECT. There is no morphological abnormality in the left parotid gland. Left parotid gland uptake seen on PET/CT was attributed to compensatory hyperactivity

Some salivary gland tumours—like adenoid cystic carcinoma, spindle cell neoplasms, extranodal marginal zone lymphomas, as well as necrotic tumours or necrotic lymph node metastases, well-differentiated sarcomas and some thyroid carcinomas—may not be FDG-avid, thereby yielding false-negative results (Fig. 14). FDG-PET/CT is therefore not recommended for evaluating these tumours. [1, 3, 4, 9, 19, 42, 65]. Low-grade tumours may show lower FDG uptake than their high-grade counterparts [20, 66]. Also, necrotic lymph nodes may not contain sufficient metabolically active tissue to show FDG uptake, even though the primary solid neoplasm may show FDG uptake. This potential pitfall must be considered to avoid false-negative results [1, 3, 4, 9, 19, 33, 42].

**Fig. 15:**
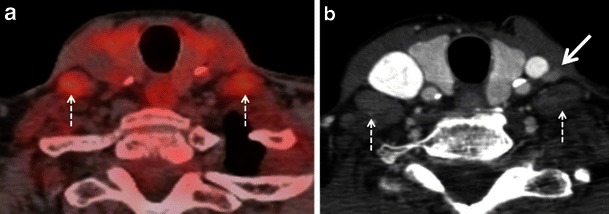
**a** Axial PET/CT image shows no evidence of abnormal FDG uptake or metastatic cervical adenopathy in the neck. Physiological mild FDG uptake is seen in the thyroid gland, oesophagus and in the scalenus muscles (*dashed arrows*). This patient was a follow-up case of a SCC of the base of the tongue. **b** Corresponding CECT image (1 mm slice) detects a 5 x 7 mm sized enhancing node at left level IV (*white arrow*). This node did not show an increased FDG uptake on the PET acquisition of the PET/CT (slice thickness of PET acquisition was 5 mm). US FNAC revealed metastatic lymphadenopathy. Neck dissection confirmed metastasis from HNSCC. Dashed arrows in b point at normal anterior scalenus muscles

Small-sized malignant tumours (diameter below 6–8 mm) and especially small-sized metastatic lymph nodes may not be detected by PET/CT unless intense FDG uptake is present because these lesions are below the resolution of current PET scanners (Fig. 15). Partial volume effect may cause significant decrease in perceived SUV in such small lesions and thereby yield a false-negative result. This pitfall can be partly overcome today by using dedicated HR PET/CT acquisitions (see above), whereas future developments in PET detector technology hold promise to further improve scanner resolution [1, 4, 9, 33, 42, 67].

#### Lesion proximity to areas with high metabolism

**Fig. 16:**
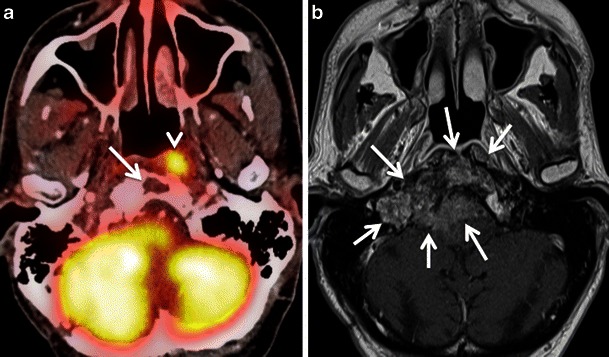
**a** Axial PET/CT image shows asymmetric uptake in the nasopharynx (arrowhead) and expected high uptake in the explored hindbrain. A lytic lesion in the clivus with well-defined sclerotic borders and without FDG uptake is also detected (*arrow*). The remaining of the total body PET/CT was normal. **b** Corresponding axial contrast-enhanced T1weighted MR image obtained in the same patient illustrates an infiltrative, poorly delineated tumour invading the clivus, the right jugular fossa, the right petrous apex and the brainstem (*arrows*), not revealed by PET/CT. Subsequent biopsy of the clivus, intracranially and of the nasopharynx showed a primary adenocarcinoma of the skull base. The increased FDG uptake in the left nasopharynx corresponds to tumour invasion of the longus colli muscle. Due to intratumoral areas with variable FDG avidity and due to tumor vicinity to the highly metabolic brain parenchyma, this lesion is less well depicted by PET/CT than MRI

**Fig. 17:**
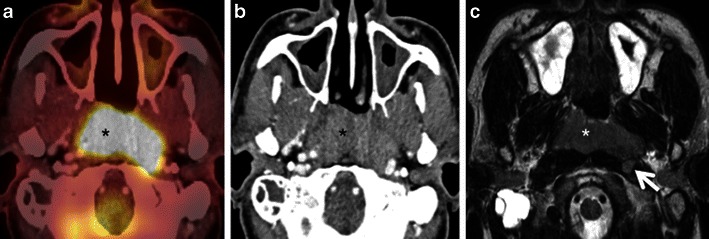
**a** Axial PET/CT image reveals a FDG-avid mass in the nasopharynx in keeping with a known nasopharyngeal carcinoma (*asterisk*). No separate retropharyngeal adenopathy is seen on this image. **b** Corresponding axial CECT image shows the infiltrative nasopharyngeal carcinoma (*asterisk*). No definite retropharyngeal adenopathy is seen. **c** Corresponding axial T2W MR image demonstrates the infiltrative nasopharyngeal carcinoma (*asterisk*) and an enlarged left retropharyngeal node (*white arrow*) suspicious for nodal metastasis. This node was missed on PET/CT due to its close proximity to the FDG-avid primary tumour and on CECT due to poor soft tissue contrast as compared to MRI. Detection of retropharyngeal lymph nodes in nasopharyngeal cancer affects TNM classification

Proximity of a pathological lesion to another FDG-avid lesion or normal structure with high FDG uptake may lead to a false-negative diagnosis. This is most commonly seen in skull base tumours in the vicinity of highly metabolic brain parenchyma or in small oral cavity tumours close to the tonsils, which show high physiological FDG uptake (see above) [3, 4, 9, 33, 68]. In the orbit, the high metabolic activity of extraocular muscles acts as a further confounding factor [69]. In addition, abnormalities of the skull base and orbit tend to be overlooked on head and neck PET/CT examinations also because they are often subtle and they are typically at the edge of the field of view [69]. Detailed anatomic evaluation of the CT part of PET/CT is essential for the detection of subtle obscuration of fat planes beneath the skull base or bony erosion of skull-base foramina. Nevertheless, an additional MRI examination (Fig. 16) is often necessary for the precise evaluation of tumour spread in these specific regions. A common example of this pitfall occurs in the evaluation of primary nasopharyngeal carcinoma with FDG-PET/CT. FDG PET/CT is known to underestimate perineural spread, tumour involvement of the skull base and cavernous sinuses compared with MRI, due to the surrounding ‘shine through effect of FDG’ around the tumour and because of the inferior conspicuity of these conditions on CECT compared with MRI [4, 33, 70]. However, the higher FDG accumulation in the tumour as compared to that in the adjacent grey matter and changing the window and level settings on PET/CT scans may help to pick up the lesion in some cases [48, 49]. Due to the close vicinity of retropharyngeal lymph nodes to the nasopharynx, detection of retropharyngeal nodal metastases with PET/CT may be impossible and correlation with MRI is necessary for correct tumour staging [4, 33, 70] (Fig. 17). The recent introduction of hybrid PET/MRI systems is expected to facilitate image interpretation in these particular situations [5, 48–51]. Boss et al. have shown promising results for the evaluation of skull base and suprahyoid neck tumours using PET/MRI hybrid systems [49], and Vargas et al. [50] and Varoquaux et al. [51] reported excellent PET/MRI image quality and lesion conspicuity in head and neck cancer patients. Nevertheless, PET/MRI systems are still not widely available [5] and ongoing research will clarify potential future applications in the head and neck.

### Common FDG-PET/CT artefacts

#### Artefacts related to dental hardware and metallic implants

**Fig. 18:**
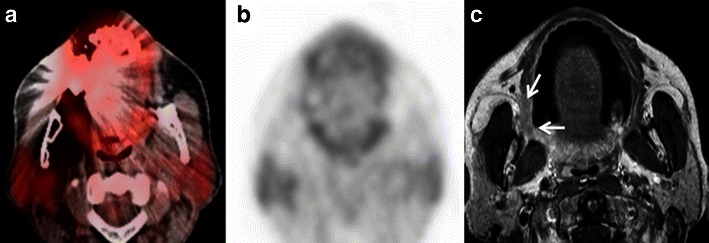
**a** Axial PET/CT image demonstrates extensive streak artefacts from right-sided dental implants in a patient with clinically proven SCCof the right retromolar trigone. The known lesion in the right retromolar trigone is completely obscured by the streak artefacts. **b** Corresponding axial FDG-PET image depicts no uptake in the region of the right retromolar trigone thereby yielding a false-negative result. **c** Corresponding axial contrast-enhanced T1W MR image detects the infiltrative mass in the right retromolar trigone (*white arrows*). The extent of the lesion as seen on MRI was afterwards confirmed surgically. MRI is less affected by dental artefacts as compared to CT

Unremovable dental hardware and metallic implants are a major problem in cross-sectional imaging of the head and neck. They can severely degrade the visual appearance of CT and CECT images by causing streak artefacts and affecting the true distribution of Hounsfield units. These artefacts may propagate to the PET images through CT-based attenuation correction factors [2, 71]. Attenuation-correction artefacts may negate the utility of CT for the spatial localisation of PET findings. Because artefacts related to metal implants are in general less pronounced on MRI scans, a combined interpretation of PET/CT and MRI helps to avoid false-negative readings and/or inaccurate tumour assessment (Fig. 18). Artefacts related to dental hardware and metallic implants also affect measurements of SUV values on PET/CT [72]: while SUVs tend to decrease in the dark streak artefact regions, they increase significantly in the bright streak artefact regions [73]. Appropriate algorithms for the correction of metallic artefacts on CT help to overcome this pitfall [72]. These algorithms help to suppress the bright and dark streak artefacts, thereby increasing the HUs in areas where values have been underestimated and decreasing the HUs in areas where values have been overestimated. Inspection of the emission data (non-attenuation-corrected PET scan) often helps to resolve any uncertainty regarding the presence of a CT-based attenuation artefact [2, 6, 74]. Recently, it has been suggested that dental streak artefacts can be corrected during CT-based attenuation correction using complementary MRI data [75].

#### Patient motion

Involuntary patient motion between the CT and the PET data acquisition of a PET/CT study can lead to poor data fusion, making correct localisation of focal uptake impossible especially in smaller lesions and in the supraclavicular area (see “Increased FDG thyroid gland uptake”). In addition, respiratory mismatch or vigorous swallowing can also influence the quality of PET/CT images as head and neck cancer patients often present with major dyspnoea, coughing or swallowing problems. In order to optimise PET/CT image fusion, the respiratory levels during the PET and CT data acquisition should be similar. Therefore, in most institutions, patients are asked to breathe in a shallow fashion during the neck acquisition and refrain from vigorous inspiratory or expiratory manoeuvres. In addition to adequate patient instruction, immobilisation during scanning often prevents motion artefacts. Nevertheless, it is important to point out that mis-coregistration artefacts from patient motion are a lesser problem in head and neck PET/CT studies compared with PET/CT scans of the chest or abdomen.

## Conclusions

FDG-PET/CT imaging has dramatically changed head and neck cancer imaging and management. FDG, however, is not tumour-specific and various image interpretation pitfalls may occur due to false-positive and false-negative causes of FDG uptake. This article reviews the causes of physiological FDG uptake in the head and neck, and of uncommon patterns of uptake due to benign lesions, artefacts, recent surgery, inflammation and scarring. Awareness of these tumour mimics along with accurate clinical information, detailed anatomical evaluation and—if necessary—correlation with other imaging modalities, such as CECT, US or MRI help to avoid misinterpretation of head and neck PET/CT studies.
